# Using transrectal ultrasound to examine the effect of exogenous progesterone on early embryonic loss in sheep

**DOI:** 10.1371/journal.pone.0183659

**Published:** 2017-08-25

**Authors:** Jessica Paige Rickard, Gabrielle Ryan, Evelyn Hall, Simon Paul de Graaf, Robert Hermes

**Affiliations:** 1 Faculty of Science, School of Life and Environmental Sciences, The University of Sydney, NSW, Sydney, Australia; 2 Department of Reproduction Management, Leibniz Institute for Zoo and Wildlife Research, Berlin, Germany; University of Florida, UNITED STATES

## Abstract

The financial impact of early embryonic loss in Australia may be as high as $137 million AUD/year. Embryos may be lost due to environmental conditions, or maternal factors such as nutrition or progesterone (P4) profiles. However, studies on the supplementation of P4 during early pregnancy have returned contradictory results, partly as a reliable method of detecting embryos in the early stages of gestation (<day 20) has yet be established. As such, Merino ewes (n = 62) were either not supplemented (control) or were given exogenous P4 at the time of insemination (day 0) or 3 days later (day 3). Transrectal ultrasound (TRUS) was performed on day 10, 12, 14, 17, 19 and 29 following laparoscopic artificial insemination. Transcutaneous ultrasound (TCUS) was performed on day 54 to confirm pregnancy and peripheral blood was collected for hormone analysis on day 19 to compare the accuracy of all three pregnancy diagnosis methods. Data were then analysed in developmental periods. The percentage of ewes detected as pregnant by TRUS during pre-, peri- and post implantation was 66% (41/62; day 12 and 14), 61% (38/62; day 17 and 19) and 58% (36/62; day 29), respectively. TCUS during established gestation recorded a pregnancy rate of 60% (37/62). The sensitivity of TRUS to correctly diagnose ewes as pregnant during pre-, peri- and post implantation was 68% (25/37), 89% (33/37) and 100% (36/36), respectively, while the sensitivity to correctly identify multiples was 49% (16/33), 60% (21/35) and 97% (34/35), respectively (P<0.05). The majority of embryonic loss occurred between pre- and peri- implantation (0.9±0.15 per ewe; P<0.001). No further loss was recorded after this point. Ewes that were given P4 at day 0 had significantly higher embryonic loss (77%) compared to the control (52%) and day 3-ewes (56%; P<0.05). These results show TRUS is a viable tool for investigating early embryonic loss and that the variability noted in previous P4 supplementation studies may be due to variation in time and length of treatment.

## Introduction

Early embryonic loss in sheep is a major source of reproductive wastage, accounting for significant economic losses to the industry. In ewes, it is estimated that around 20–30% of all fertilised ova [1] will succumb to mortality and despite attracting considerable research attention, the causative agents, exact time of loss and methods of prevention have yet to be fully determined. Working on slaughter numbers calculated for Australia in 2015 [2] and the current market value per average carcass weight at slaughter [3], these “lost lambs” may amount to a financial loss of up to $137 million AUD/year. A major hurdle for investigating this early embryo mortality is the ability to detect embryos in the very early stages of gestation. Therefore, before a means of prevention can be established or tested, a suitable, accurate and reliable method for detecting pregnancy as well as number of conceptus’ per ewe prior to the industry standard of day 54 (real time cutaneous ultrasound) must to be developed.

The current technique for exploring early embryonic loss utilises ovulation rate, which is often used as a proxy for conceptus number. This value is then compared to traditional real-time ultrasound pregnancy diagnosis or lambing percentage [4]. This is a logical substitution for embryonic loss, due to the fact that sheep are generally believed to hold a high fertilisation rate and scarcely undergo late gestational losses [5]. However, this method does not represent accurate embryo loss data, especially considering that literature predicts the majority of loss to occur in sheep prior to implantation, which occurs at approximately day 14–16 [6, 7]. If embryos could be detected during early pregnancy (before or shortly after implantation), a more thorough and unambiguous means of studying early embryonic loss could not only be established but methods of prevention could be better evaluated.

While previous studies [8–12] have utilised transrectal ultrasound (TRUS) to detect pregnancy in sheep, limited work has shown a high accuracy of TRUS earlier than day 18 and no study to our knowledge has attempted to identify multiples at this stage of pregnancy. The earliest Romano and Christians (10) detected pregnancy was day 16, recording a pregnancy rate of 27% (8/30) with an accuracy of 31%. We hypothesise that if an ultrasound transducer with a higher resolution were used, pregnancy could be detected earlier than day 16 and this would be accompanied by higher accuracy.

With the development and refinement of an advanced ultrasonography technique, not only could we begin to examine the causes of embryonic loss but also investigate ways to reduce it. Maternal factors such as nutrition [12, 13], age [14] and genetics [15] of the ewe, as well as endocrine factors [6, 16] and environmental conditions, such as heat stress and hypothermia [17] have all been linked to early embryonic loss in sheep. In particular, variance in circulating concentrations of progesterone (P4) during early pregnancy (<day 30) has been judged to contribute to early embryonic loss [11, 16, 18–24]. Parr et al. [13] investigated the influence of plasma P4 levels on embryonic survival and reported an inverse relationship between decreasing mortality at day 11 and 21 post mating and increasing P4 supplementation. Simarily, Ashworth et al. [25] and later in 1989 [26] as well as O’Connell et al. [21], reported increased embryonic loss in ewes with lower P4 levels at Day 4–5. The reported benefit of P4 supplementation is hardly suprising given its reputed importance in preparation of the uterus for embryo development, maternal recognition and implantation [27, 28]. However, several studies have found no effect [16, 20] with some even reporting increased embryonic loss when P4 supplementation was given on Day 1 following mating [29]. These contradictory findings highlight the necessity of further research into the viability of exogenous P4 supplementation to prevent embryo loss, particularly utilising advanced ultrasonography methods.

As such, the aims of the present study were to establish the earliest point of gestation transrectal ultrasound can accurately detect pregnancy in sheep and observe whether exogenous P4 supplementation during early pregnancy reduces the incidence of early embryonic loss.

## Materials and methods

### Experimental design

This study was carried out in strict accordance with the Australian Research Act 1985 No. 123 and the Australian code for the care and use of animals for scientific purposes 8^th^ edition (2013). Procedures herein were approved by the University of Sydney’s Animal Ethics Committee (Protocol number: 2016/965). All procedures were performed by experienced technicians or veterinarians and all efforts were made to minimise suffering. Unless otherwise stated all chemicals were supplied by Sigma-Aldrich (St Louis, MO, USA).

Following synchronisation, Merino ewes underwent laparoscopic intrauterine insemination 55 hours post progestogen sponge removal. Immediately following insemination, ewes were divided into three treatment groups based on exogenous progesterone supplementation (Eazi-Breed Controlled Intravaginal Drug Release (CIDR); Zoetis, Rhodes, Australia); no exogenous progesterone administered (No CIDR; control; n = 21 ewes), exogenous progesterone administered post insemination (CIDR at Day 0; n = 20 ewes) and exogenous progesterone administered on Day three post insemination (CIDR at Day 3; n = 21 ewes). CIDRs were removed on Day 17 post insemination (1 cycle). Ewes underwent transrectal ultrasound on Day 10, 12, 14, 17, 19 and 29 post insemination. Peripheral blood plasma was obtained for progesterone hormone analysis on Day 19. Transcutaneous ultrasound was conducted on Day 54 to confirm pregnancy diagnosis.

### Animals

Rams (3 yrs old; body condition score 3–4) and mature multiparous Merino ewes (n = 62; 2–4 yrs old; body condition score 2–3) were kept on a pasture-based diet supplemented with whole cracked lupins ad-libitum at the Cobbitty Sheep and Horse Unit, Faculty of Science, School of Life and Environmental Sciences, University of Sydney, Camden, NSW, Australia. All animals were assessed for body condition score as per industry practise [30].

### Collection and preparation of semen for insemination

Semen was collected from a mature Coopworth ram (n = 1 ram x 2 ejaculates combined) using an artificial vagina, in the presence of a teaser ewe. Ejaculates were immediately assessed for wave motion as per industry standard with only ejaculates scoring 4 or higher accepted for insemination [31]. Two ejaculates were combined and concentration of the pooled semen determined using a SDM1 photometer calibrated for ram semen (Minitube; VIC, Australia) and slowly extended to 600×10^6^ spermatozoa/mL with a warmed (37°C) tris, citrate, fructose solution, supplemented with 15% egg yolk [31]. Samples were maintained at 30°C until laparoscopic insemination. The motility of spermatozoa was regularly assessed (subjectively) [31] to ensure high motility prior to insemination.

### Oestrus synchronisation and laparoscopic insemination

Mature Merino ewes (n = 71) were synchronised for oestrus as per industry practice, using a combination of intravaginal progestagen-impregnanted sponges (30 mg Flugestone Acetate; Vetoquinol; Brisbane, Australia) for 13 days followed by an intramuscular injection of 400 IU PMSG (1 mL Pregnecol; Vetoquinol; Brisbane, Australia) at sponge removal. All ewes were fasted 24 hours prior to insemination and were left undisturbed for approximately 3 hours following insemination.

Ewes were inseminated by intrauterine laparoscopy, 55 hours post sponge removal with 50×10^6^ motile spermatozoa/ewe (insemination volume of 0.1 mL) using standard industry techniques [31]. Ewes were inseminated with semen from the same ram (and combined ejaculate) to avoid variation in the effect of sire on prenatal embryo loss and prolificacy in ewes, as discussed by Holler et al. [32] and Carr et al. [33]. Ewes were sedated and given pre-operative analgesia via an intramuscular injection of Xylazil (5 mg; Ilium Troy; Glendenning, Australia).

### Pregnancy diagnosis

#### Transrectal ultrasound (TRUS)

**Fig 1:**
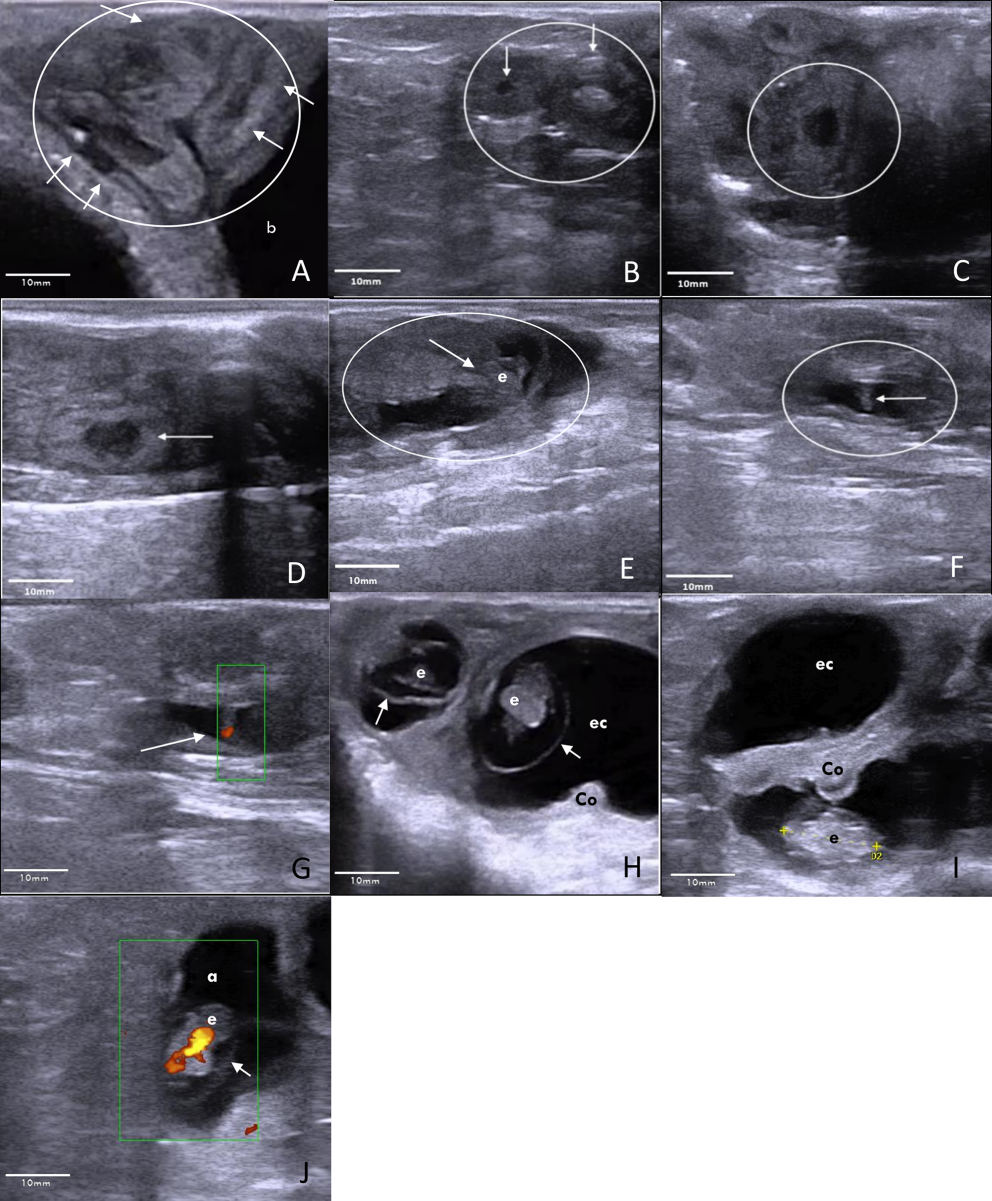
Transrectal ultrasound (Esaote Germany, MyLab™One VET, equipped with electronic linear array 10–5 MHz transducer (SV3513 Vet)) images of the ovary, uterus and developing conceptus during early pregnancy in sheep (oestrus = day 0). A Day 10: The coiled uterus (circle) is located cranial to the bladder (b). Within the coiled uterine horns, the lower echogenic endometrium (arrows) is well distinguished. B Day 10: Two corpora lutea (arrows) are present on the ovary (circle), documenting recent ovulation. C Day 12: Trophoblastic expansion (within the circle): At the site of trophoblastic expansion the endometrium appeared enlarged. A dark oval shaped structure expanded within the uterine lumen. Boundaries between endometrium and trophoblast could not be distinguished at this stage. Yet the trophoblast expanded the uterine lumen exceeding the regular endometrial height by 2-fold. D Day 14: Trophoblastic expansion (arrow): Low echogenic oval-shaped trophoblast within the uterine lumen, further expands in length but not in height. E Day 17: Embryonic vesicle (circle) with embryo (arrow) within. F Day 19: Embryo (arrow), distinctly present inside the embryonic vesicle (circle). G Day 19: Power Doppler of the embryo (arrow) heartbeat. H Day 29: Two embryo’s (e) surrounded by the amniotic membranes (arrows). A cotyledon is in close proximity of the embryo (Co). Crown rump length of the embryo measures 14 mm. Fluids of the embryonic cavity (ec) expand further into the adjacent uterine horn. I Day 29: Embryo (e), situated below a cotyledon (Co). Above this the fluid filled embryonic cavity (ec), which expands into the uterine horn. J Day 29: Power Doppler of the embryo heart. Embryo (e) is surrounded by the amnion (a) and the fluid filled embryonic cavity (arrow).

Ewes were given an enema immediately prior to examination to remove faeces from the rectum before being restrained in dorsal recumbency. All ewes undergoing TRUS (Esaote Germany, MyLab™ One VET, equipped with linear array 10–5 MHz transducer (SV3513 Vet) was examined by the same veterinarian. Ovulation rates were determined by counting the number of corpora lutea on day 10 (Fig 1B). Pregnancy (including number of multiples) was diagnosed based on the appearance of trophoblast expansion areas (day 12 and 14; Pre-Implantation; Fig 1C and 1D), embryonic vesicles (day 17, 19 and 29; peri and post-implantation; Fig 1E and 1F) and embryos (Day 17, 19 and 29; peri and post-implantation; Fig 1G, 1H and 1I)

#### Blood hormone analysis

Pregnancy status was also determined during peri-implantation (day 17 and 19) by analysis of progesterone concentration as per manufacturer’s instructions (ImmuChem™ Coated Tube 125 RIA Kit; MP Biomedicals; Costa Mesa, CA, USA; CV <10%, sensitivity; 0.02ng/mL) following collection of jugular blood samples (5-10mL). Heparin treated blood samples were centrifuged (3 000 ×g; 15 mins; room temp) before being frozen at -20°C. Ewes with progesterone concentrations >1ng/mL were considered pregnant [34]. To ensure the exogenous progesterone supplementation did not interfere with the ewes’ endogenous progesterone production, blood samples were taken 24 hours after CIDR removal (as per the recommended withholding period; Zoetis, Australia).

#### Transcutaneous ultrasound (TCUS)

Ewes were also scanned via transcutaneous ultrasound (Ovi-Scan 6; Axial 3.5MHz transducer; BCF Ultrasound Australasia; Mitcham, Australia) as per manufacturer’s instructions, 54 days post insemination to confirm pregnancy diagnosis and conceptus number.

### Data and statistical analysis

#### Percent pregnant by TRUS

TRUS scanning days were broken up into 5 main developmental periods and embryonic loss over time was compared accordingly; Ovulation: a measure of the number of corpora lutea present on the ovaries of each ewe on day 10 and 12; Pre-Implantation: a measure of the number of trophoblast expansions within the uterine endometrium on days 12 and 14, Peri-Implantation: the number of the embryonic vesicles on days 17 and 19, Post-Implantation: the number of embryos on day 29 and Established gestation: the number of conceptus’ detected on day 54. To determine whether there was a significant difference between the number of pregnant ewes detected during each developmental period, data were analysed using a Generalised Linear Mixed Model (GLMM) with an underlying binomial distribution in GenStat (Version 15, VSN International, Hemel Hempstead, UK), with period as a fixed effect and ewe tag as a random effect.

#### Accuracy of pregnancy diagnosis by TRUS

The data recorded via TRUS during pre-implantation (day 12/14), peri-implantation (day 17 and 19) and post-implantation (day 29) were compared to the data recorded via TCUS during established gestation (day 54). If a ewe was recorded as pregnant during TRUS and this was confirmed by TCUS on day 54, the result was classified as a true positive (TP). If a ewe was not detected as pregnant during TRUS but was detected as pregnant via TCUS, the result was classified as a false negative (FN). Pregnancies that were detected during TRUS scanning days but not via TCUS were classified as false positives (FP) and ewes that were deemed not pregnant by both TRUS and TCUS were classified as true negatives (TN). These data were analysed using an Ordinal Logistic Regression (ORL) in GenStat, to establish any significant differences between the predicted ratios of each category (TP, FP, TN, FN) on each TRUS day. The accuracy of detecting multiples was calculated in the same way, comparing data recorded by TRUS to TCUS on day 54. The sensitivity of using TRUS to correctly and accurately detect pregnancy as well as multiples per ewe was determined by calculating the percentage of TP over the sum of TP and FN during each developmental period as described by Moufort and Miller (35).

Similarly, the accuracy (using day 54 as the standard) associated with detecting pregnancy during peri-implantation via TRUS was calculated and compared to the accuracy associated with P4 blood hormone pregnancy analysis in the same way as described above. Further, a Restricted Maximum Likelihood (REML) model in GenStat, with the fixed effect of treatment and random effect of ewe was used to determine whether there were any significant differences between P4 blood plasma concentrations across treatments.

#### Determination of embryonic loss over time

Only ewes that were identified as pregnant during pre-implantation were included in the embryonic loss analysis. The total number of conceptuses that were lost over the entire scanning period were tallied and then averaged per ewe. A Restricted Maximum Likelihood model (REML; GenStat) was used on the raw data to determine whether the number of conceptuses lost was significantly different to total ovulation rate per ewe. Ewe tag was defined as the random model, while period was defined as the fixed model.

A REML was also used in GenStat to determine whether the number of conceptuses lost between each developmental period was significantly different and whether there was an effect of exogenous P4 supplementation treatment (no P4, P4 at day 0, P4 at day 3). Period and treatment were included as fixed effects, with the random effect of ewe tag. The analysis was run on raw values that had previously been standardised as a percent of ovulation rate.

#### Ovulation, embryonic vesicle and embryo characteristics

The length of each corpus luteum, embryonic vesicle and embryo was measured using the Esaote Germany, MyLab™ One VET software. Raw values were compared over time and between exogenous progesterone treatments using a restricted maximum likelihood model (REML; GenStat). Treatment and day were specified as the fixed effect, while ewe tag was specified as the random effect.

For all statistical analyses, significance was set at P<0.05.

## Results

### Detection of pregnancy and accuracy of diagnosis

**Fig 2:**
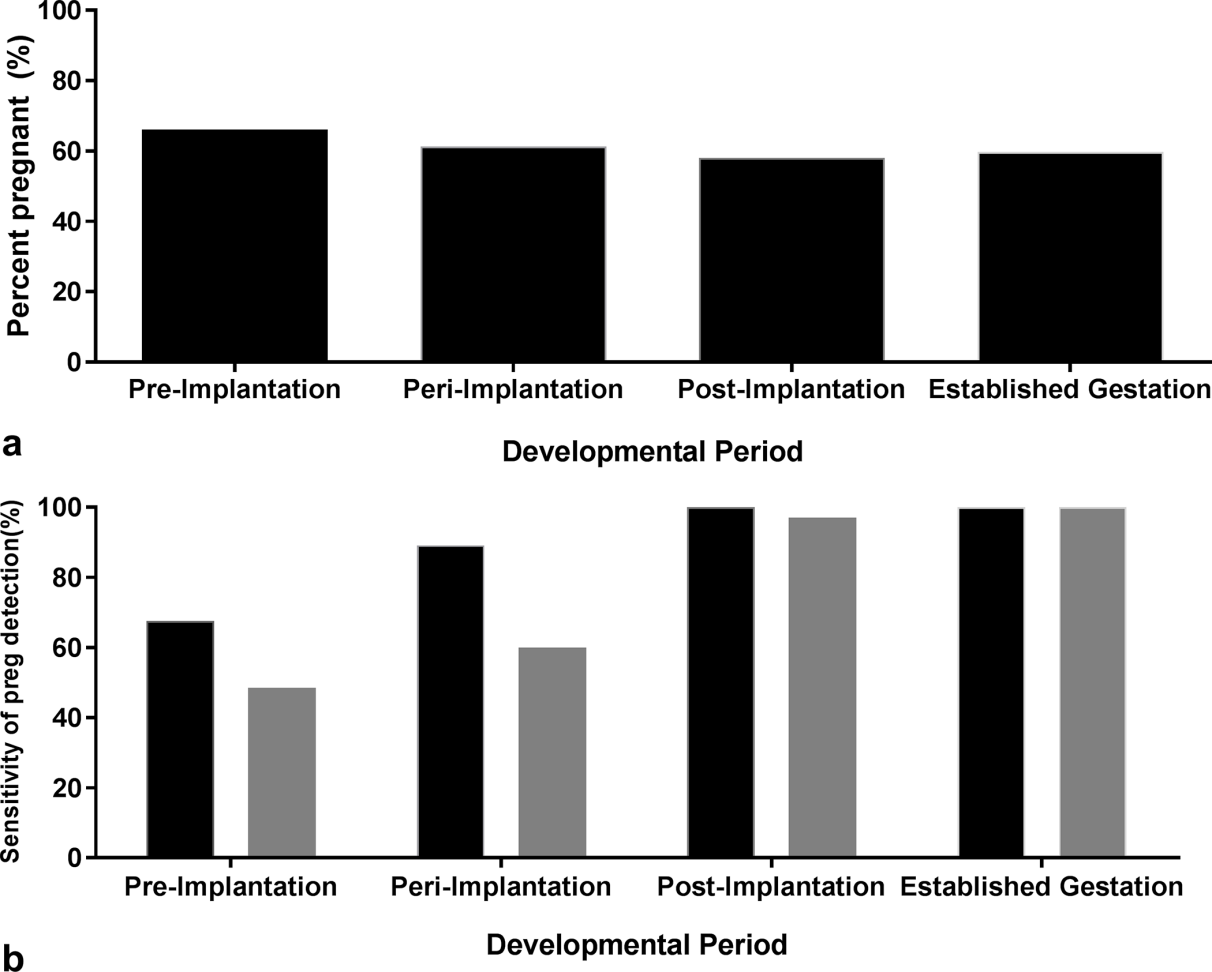
The a) percentage of ewes detected as pregnant by TRUS and TCUS (established gestation only and b) sensitivity of TRUS (TP% = #TP/#TP+#FP; [35]) to correctly detect pregnancy (black bars) and number of conceptuses per ewe (grey bars) during pre-implantation (day 12/14), peri-implantation (day 17/19), post implantation (day 29) and established gestation (day 54).

The percentage of ewes detected as pregnant by TRUS during pre-implantation (day 12 and 14), peri-implantation (day 17 and 19) and post-implantation was 66% (41/62), 61% (38/62) and 58% (36/62), respectively (Fig 2A). The pregnancy rate determined by TCUS on day 54 or during established gestation was 60% (37/62; Fig 2A). There were no significant differences in pregnancy rate among developmental periods (P = 0.07). The pregnancy rate determined by P4 blood hormone analysis during peri-implantation (day 17/19) was 49%. The mean P4 blood plasma concentration did not differ significantly among treatments (P = 0.98).

**Fig 3:**
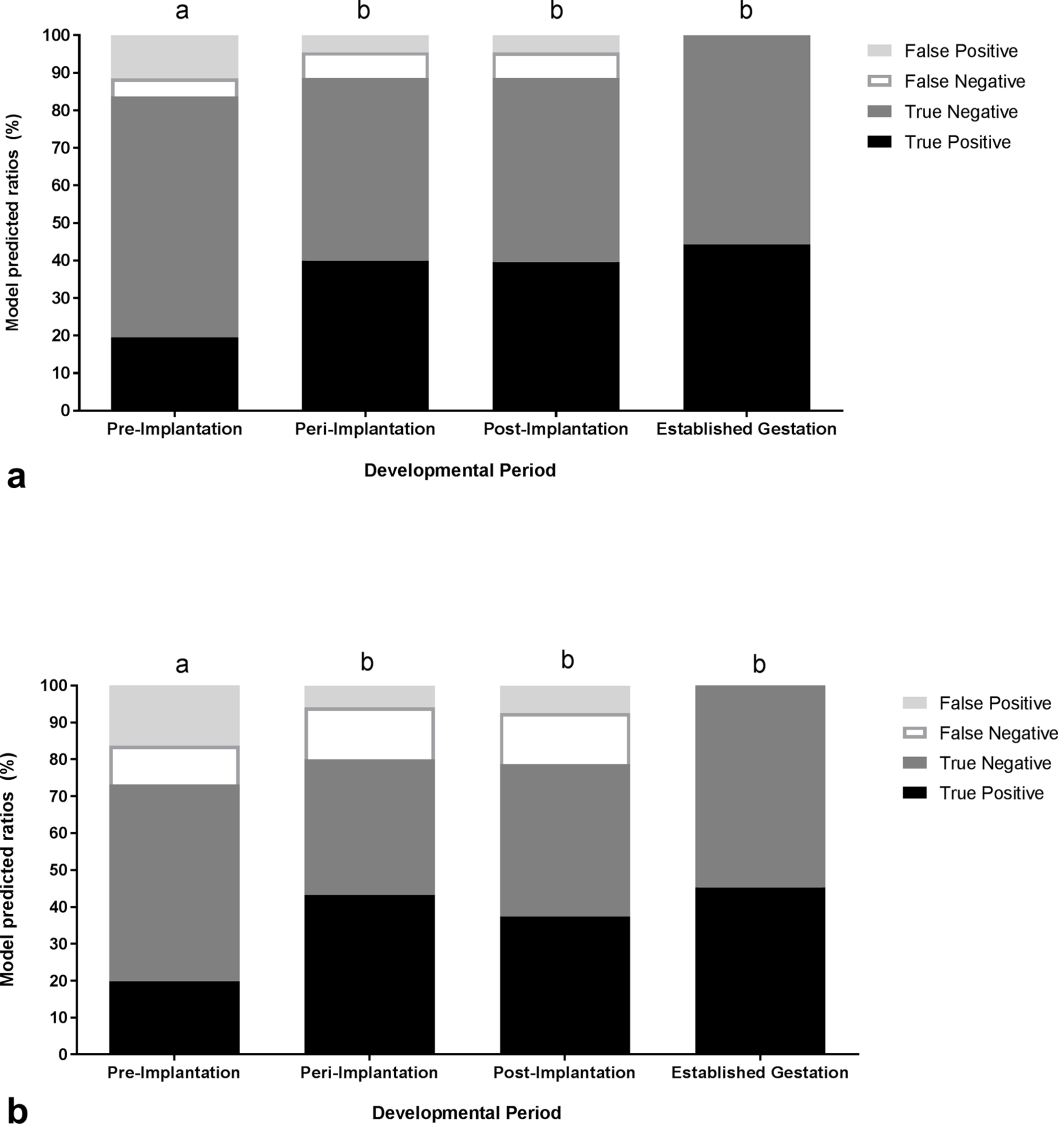
Predicted ratios of each category; true positive (black segment), true negative (dark grey segment), false negative (white segment) and false positive (light grey segment), generated from an Ordinal Logistic Regression for the accuracy of using TRUS to detect pregnancy (a) and multiples per ewe (b) during pre-implantation (day 12/14), peri-implantation (day 17/19), post-implantation (day 28) in reference to results obtained by TCUS during established gestation (day 54).

The sensitivity of TRUS to correctly diagnose ewes as pregnant during pre-implantation (day 12 and 14), peri-implantation (day 17 and 19) and post-implantation, was 68% (25/37), 89% (33/37) and 100% (36/36; Fig 2B). Similarly, the predicted ratio of accuracy categories (TP, FN, FP, TN) differed significantly during each period (P = 0.009). The number of ewes correctly identified as pregnant during pre-implantation was significantly lower compared to that identified during peri-implantation, post implantation and established gestation (P = 0.009; Fig 3A). There were no significant differences among ratios calculated during peri-implantation, post-implantation and established gestation (P = 0.98; Fig 3A).

The sensitivity of TRUS to correctly identify the number of conceptuses per ewe during pre-implantation (day 12 and 14), peri-implantation (day 17 and 19) and post-implantation, was 49% (16/33), 60% (21/35) and 97% (34/35; Fig 2B). Similarly, analysis revealed a significant difference in the predicted ratio of accuracy categories (TP, FN, FP, TN) when detecting the number of multiples per ewe over TRUS days (P = 0.005). The number of ewes correctly identified with multiples during pre-implantation was significantly lower compared to that identified during peri-implantation, post implantation and established gestation (P = 0.005; Fig 3B). There were no significant differences among ratios calculated during peri-implantation, post-implantation and established gestation (P = 0.64; Fig 3B).

When the accuracy (day 54 standard) of ewes detected as pregnant during peri-implantation via P4 blood hormone analysis (sensitivity 83%) was compared to the accuracy generated using TRUS (sensitivity 87%), there were no significant differences among pregnancy diagnosis methods (P = 0.97).

### Overall embryonic loss

**Fig 4:**
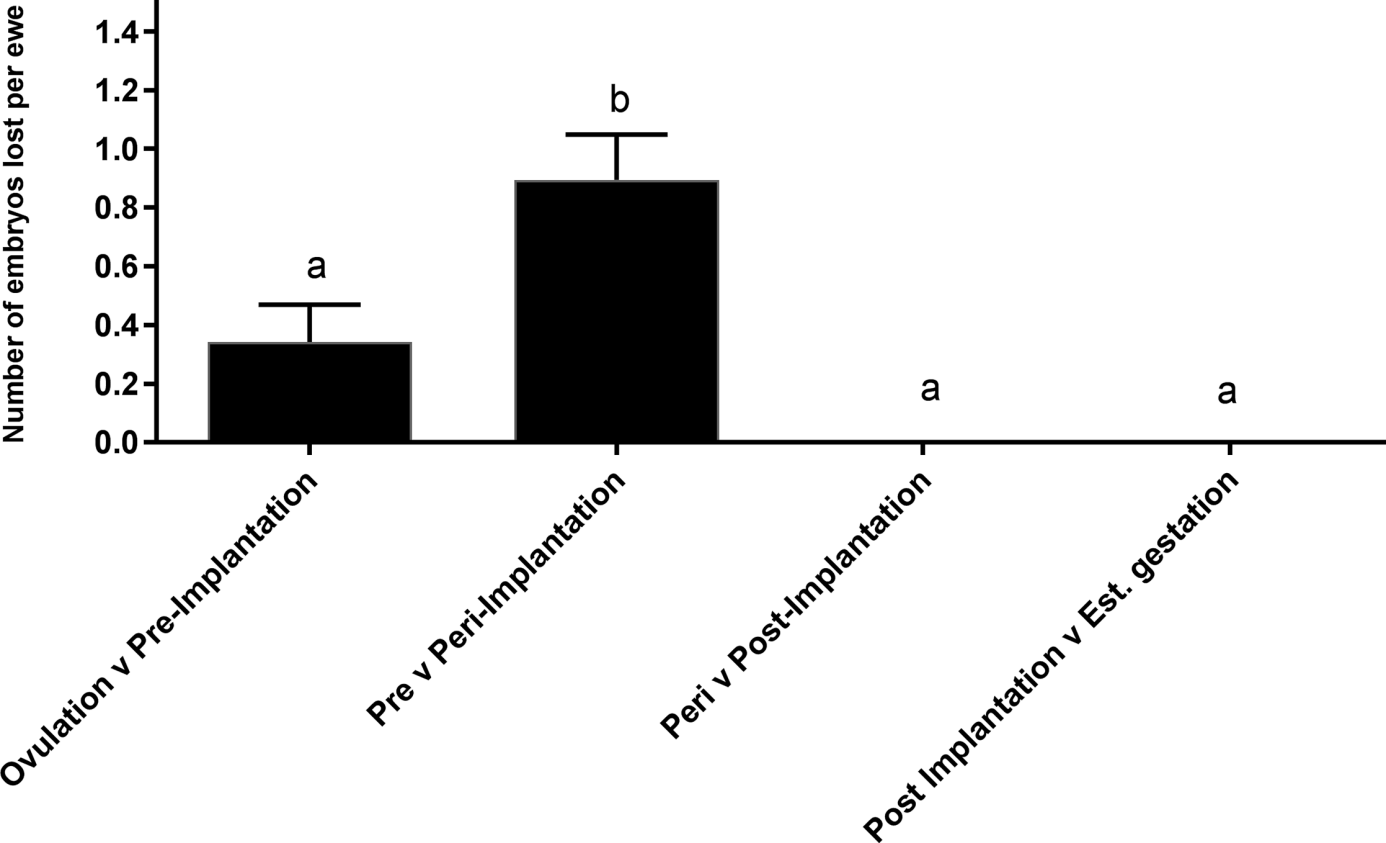
The number of embryos lost per ewe between each developmental period; ovulation v pre-implantation, pre v peri-implantation, peri v post-implantation and post implantation and established gestation. Values are mean ±SEM. Columns without common subscripts differ significantly (P<0.05).

An average of 2.1±0.11 ovulations (number of corpora lutea) were detected per ewe on day 10 and 12 of TRUS. Regardless of exogenous P4 treatment, an average number of 1.2±0.17 embryos per ewe were lost by day 54 (P<0.001). When this embryonic loss is compared across developmental periods; an average of 0.3±0.13 trophoblastic expansions per ewe were lost between ovulation and pre-implantation, 0.9±0.15 embryonic vesicles per ewe were lost between the pre and peri-implantation period, 0±0.13 embryos per ewe were lost between peri and post-implantation and 0±0.04 conceptus per ewe were lost between post-implantation and established gestation (P<0.001; Fig 4). While embryonic loss occurred up until peri-implantation, the loss detected between pre- and peri-implantation was significantly higher compared to that recorded between ovulation and pre-implantation (P<0.001; Fig 4). There was no significant difference among the embryonic loss recorded between ovulation and pre-implantation, peri- and post-implantation and post-implantation and established gestation (Fig 4).

### The effect of exogenous P4 supplementation on embryonic loss

There was no significant interaction (P = 0.09) between developmental period and treatment on embryonic loss, resulting in embryonic loss being pooled over time for each treatment. Ewes that weren’t given any additional P4 had an average ovulation rate of 2.4±0.18 and lost a total of 1.0±0.25 embryos per ewe by day 54. Ewes that were given additional P4 on day 0 post AI, recorded an ovulation rate of 1.7±0.17 and lost a total of 1.3±0.20 embryos per ewe by day 54. Ewes that were given additional P4 on day 3 post AI, recorded an ovulation rate of 2.1±0.19 and lost a total of 0.8±0.24 embryos. In order to compare embryonic loss between treatments accurately, embryonic loss was converted to a percentage loss of ovulation rate. This equates to a total embryonic loss over time of 52%, 77% and 56%, respectively. The embryonic loss recorded for ewes that received additional P4 on day 0 (77%) post AI, was significantly higher than the control ewes (that did not receive any P4; 52%) and ewes that received P4 on day 3 post AI (56%; P<0.001). There was no significant difference between the control ewes and ewes that received P4 on day 3.

### Average corpora lutea, embryonic vesicle and embryo characteristics

A total of 124 corpora lutea were detected on day 10 and 12, averaging 1.21±0.003 cm in length. There was no significant difference in size of corpora lutea between days (1.19±0.02 v 1.20±0.02; P = 0.864), nor was there an effect of treatment (1.20±0.04 v 1.17±0.06 v 1.22±±0.05, Trt 1, 2 and 3 respectively; P = 0.710).There was also no effect of treatment on the size of embryonic vesicles or embryos (P = 0.07). However, as expected a significant increase in size was observed (Day 12–29; P<0.001).

**Fig 5:**
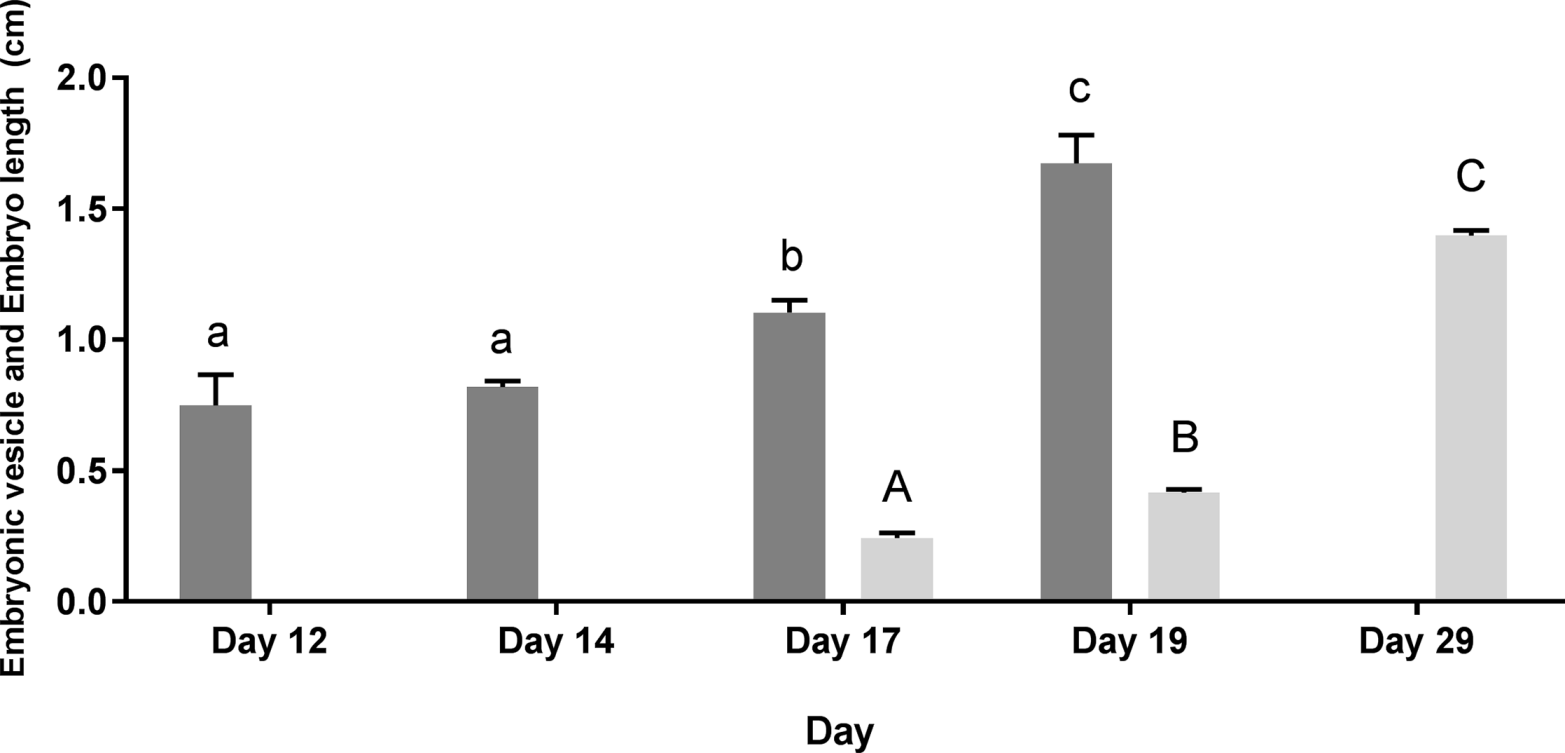
Average length of embryonic vesicles measured on day 12, 14, 17 and 19 (dark grey bars) and embryos measured on day 17, 19 and 28 (light grey bars). Values are averaged over ewe ±SEM. Columns without common superscripts differ significantly; lowercase indicates significance in relation to embryonic vesicle size, while capitalised letters indicate significance in relation to embryo size (P<0.05).

Embryonic vesicles were first detected on day 12 and averaged 0.75±0.12cm, 0.82±0.02cm, 1.10±0.05cm and 1.67±0.11cm in length on day 12, 14, 17 and 19 respectively (P<0.001; Fig 5). Embryonic vesicles on day 19 were significantly larger than those measured on day 17. Embryonic vesicles measured on day 17 were significantly larger than those measured on day 12 and 14 (P<0.001). There was no difference between day 12 and 14 (Fig 5).

Trophoblasts detected on day 12 and 14 were not stationary but moved passively within the uterine lumen. Embryos were first detected on Day 17 and averaged 0.24±0.02cm per embryo. Embryos detected on day 19 measured 0.42±0.01cm in length and were significantly larger than embryos measured on day 17. By day 29, an average crown to rump length (CRL) of 1.40±0.02cm was measured per embryo and this was significantly larger than embryos detected on day 17 and 19 (P<0.001; Fig 5).

Embryonic heartbeat was first detected on day 19 (Fig 1G) and again on day 29 (Fig 1J), in ultrasound power Doppler mode, indicating high embryo viability at the time of scanning.

Ultrasonography images of corpora lutea, embryonic vesicles and embryos with indicated measurements on each TRUS day can be seen in S1 Table.

## Discussion

The present study employed a 10MHz transducer for transrectal ultrasound (TRUS) of ewes to investigate early embryonic loss. As a result, pregnancy as well as individual embryos were detected as early as pre-implantation (day 12 and 14 of gestation). The accuracy associated with such pregnancy diagnosis during these days was also higher than previously reported [8–10]. Furthermore, the greatest period of embryonic loss in the current study was found to occur during implantation, similar to that reported by Wilmut, Sales (6). Unfortunately, these losses were unable to be mitigated by the presence of additional exogenous progesterone (P4), which even increased embryonic loss when ewes were supplemented with P4 at artificial insemination or oestrus (day 0). Such a result could be attributed to an asynchronous hormonal relationship between the uterine environment, corpora lutea and the developing embryo [18, 36].

Since the introduction of transrectal ultrasound as a method of detecting early pregnancy, the accuracy of its detection has proved pivotal to its viability as a pregnancy diagnostic and exploratory tool. Here, TRUS using a 10MHz transducer was able to detect pregnancy and the number of conceptuses per ewe during pre-implantation (day 12 and 14 following AI), recording a sensitivity of 68% and 49% respectively (Fig 2). The total number of ewes detected as pregnant remained the same over the entire scanning period, regardless of embryonic loss per ewe, however the accuracy associated with each result increased significantly over time. If going on accuracy alone, TRUS can detect pregnancy as early as day 12 and 14, but will achieve the same accuracy as that generated when using TCUS on day 54 if conducted on day 17 or 19 (peri-implantation). Nevertheless, the accuracy recorded in the current study across all developmental periods was higher than that recorded by Romano and Christians (10). While the 7.5MHz transducer used by Romano and Christians (10) failed to detect pregnancy on day 15, it recorded an accuracy of 31% on day 16 and 40% on day 17, compared to the 89% recorded in the current study using a 10MHz transducer. However, it should be noted that the number of ewes detected as pregnant on each scanning day by Romano and Christians (10) was considerably higher than that detected in the current study. We hypothesise this to be due to the difference in insemination methods, while the current study performed intrauterine laparoscopic insemination, Romano and Christians (10) used natural joining. Nonetheless, the pregnancy rates reported above are consistent with that achieved by previous fertility trials on the same property. In this study, use of a 10MHz probe, provided the ability to detect pregnancy and multiples per ewe as early as pre-implantation. While the accuracy for detecting multiples during pre and peri-implantation were up to 20% lower than that recorded for detecting pregnancy, perhaps if a higher resolution transducer (>10Mhz) was used, accuracy levels would increase. Nevertheless, these results offer an exciting new avenue for studying early embryonic loss in sheep more accurately, enabling the ability to take into account the number of conceptuses’ lost per ewe.

Adding further evidence to the accuracy of TRUS is the lack of significant difference between pregnancy diagnosis methods. There was no difference in accuracy when using P4 blood plasma concentration or TRUS on day 19. Further, there was no significant difference in the P4 blood plasma concentration between treatments, this implies that there was no lingering effect of the exogenous progesterone (CIDRs) confounding hormone assay results. The above results therefore indicate that TRUS could be adopted as a more meaningful diagnostic tool, aiding producers earlier in gestation. Currently, producers have the option of testing for P4 concentration per ewe, around day 17–19 or waiting until day 30 or 54 for TCUS. TRUS offers producers the ability to detect pregnancy as early as day 17 or 19 without the added cost of hormone assay kits. Indeed, an experienced technician is required, but the producer could draft ewes based on pregnancy status and estimated conceptus number, information that is not possible when conducting blood hormone analysis. Drafting earlier, say around day 14 or 17 could mean pregnant ewes, including those diagnosed with twins or triplets would be exposed to a more suitable nutrition profile sooner specifically during the most sensitive peri-implantation period, potentially improving embryonic development and lamb birth weight [37]. Further study on whether an amended nutrition profile earlier in gestation would ameliorate embryonic loss is definitely warranted.

The losses incurred by producers during early embryonic development in sheep represents considerable reproductive potential. In the current study, TRUS detected no further loss after day 19 (peri-implantation), indicating that the critical period for embryonic loss was from fertilisation to shortly after implantation. Indeed, low levels of prenatal loss have been reported to occur throughout ovine gestation [11, 32], yet they were not detected in this study. Nevertheless, these results agree with previous studies in sheep, who report that the majority of embryonic loss occurs prior to day 30 [1, 6, 18, 38], with most loss concentrated to the period immediately preceding day 19 [39]. Implantation in the ewe is generally perceived to occur between days 14–16 [7, 27, 40–42] but has been reported to occur as early as Day 10 [43] and as late as day 18 [44]. Several factors both maternal and embryonic have been postulated to increase the risk of mortality during this pre-and peri-implantation period. Embryos might be abnormal due to inherited defects during fertilisation and meiosis or there might be an asynchronous relationship between the embryo and uterus. This could be due to an unsupportive maternal environment [6] owing to imbalanced hormone levels [6, 16, 26], maternal nutrition [12, 13] or age [14]. In regards to the current study, it is likely that a combination of these factors are at play, increasing consideration of the idea that the quality of the oocyte and its developmental potential plays a more pivotal role in influencing the success of early embryonic development [38, 45]. Often fertilisation is achieved, yet errors or lethal effects do not occur until downstream, such as during implantation. Unfortunately in the current study, it wasn’t possible to cull ewes and collect embryos following identification by TRUS, as this would have provided a more detailed comparison of the characteristics of viable and non–viable embryos. Fortunately, O’Connell et al, [38] described such a comparison when they saw an embryonic loss of 12% during day 4–14 of gestation. Here the author hypothesises that the adhesive or signalling properties of degenerate embryos could differ from viable ones and could therefore be subjected to high rates of phagocytosis by the maternal immune system [38]. In a more applied study, Bari et al, [46] reported that during embryo transfer, grade 1 and 2 embryos survived significantly better than grade 3 or 4 embryos, again confirming that successful implantation is not solely influenced by the maternal environment.

Nonetheless, studies over the past 35 years have tried to understand the uterine relationship between maternal nutrition and unbalanced progesterone levels on early embryo mortality. Malnutrition both prior to and following joining has been linked to increased ova wastage [47–49] and a reduced secretion of IFN-T in vitro [50]. On the other hand, ewes fed above maintenance diets post insemination have returned lower pregnancy rates than undernourished ewes, thought to be due to an increased metabolic clearance rate of progesterone [51, 52]. The ewes in the current study were of similar body condition score and fed similar pasture, so the difference noted between treatments is unlikely to be caused by nutrition. Given the intricate balance of hormones during pre and peri-implantation, it is hardly surprising that an imbalance in progesterone and other reproductive hormones is often blamed for embryo mortality. Such conclusions have also been reported in cattle [4, 53, 54], pigs [55] and horses [56] and have warranted continued investigation into the effect of exogenous P4 supplementation on early pregnancy survival.

Despite considerable investigation, exogenous progesterone supplementation as a means for preventing embryonic loss is still inconclusive. In the present study, the provision of progesterone at the time of insemination proved counterproductive to embryonic survival. Ewes that received P4 at the time of insemination recorded higher embryonic loss than both the control ewes (that didn’t receive any P4) as well as ewes that received P4 on day 3 following insemination. These results agree with those reported by Kleeman et al, [29], who did not see any effect of P4 when provided to ewes on day 3–6 after ovulation but saw a decrease in pregnancy rate when provided on day 1. Here the authors suggest that this increased loss is due to an asynchronous relationship between the embryo and the uterine environment [29]. Previous literature has documented the effects of additional progesterone during early gestation, including; the acceleration of embryos from the oviduct into the uterus (pig [57]) and an increased production of polypeptides, bovine trophoblast proteins and endometrial secretory proteins (cow and sheep [26, 58]). In regards to the embryo itself, progesterone treated- embryos are more likely to develop faster, contain a higher presence of macrophages and have fewer blastomeres [29]. Furthermore, it has been shown in sheep that P4 exposure early in the oestrus cycle causes a release of prostaglandin, which initiated premature luteolysis of the corpus luteum and a shortening of the natural cycle [22–24, 36, 54, 59], ensuring flock synchrony for controlled breeding programs. This same mechanism could have caused the increased embryonic loss seen in Day-0 ewes. The additional progesterone caused premature luteolysis of the ovulatory corpus luteum halting the natural secretion of P4, but it presence was also able to maintain embryonic development until the CIDR providing the P4 was removed. These authors concluded that progesterone regulates the ability of the uterus to secrete prostaglandin in response to a blastocyst or embryo of appropriate age. This holds true for the other studies investigating the effect of progesterone on early embryonic loss. Studies in sheep that found a detrimental or lack of effect on embryonic survival, administered treatment (silastic implants or CIDRs) on day 6 [16] or day 4 following mating [20], while those that saw a beneficial effect, administered treatment as late as day 11 and 21 after mating, seeing an increase in survival with increasing doses of progesterone (5-25mg/day) [13]. Clearly, the influence of exogenous progesterone on embryonic survival is intricately related to the time of administration and stage of embryonic development.

This study was able to document the early growth and development of the ovine embryo, in vivo, using TRUS. Using a 10MHz probe, TRUS was able to identify the embryonic disc on day 12 and the developing embryo on day 17 post AI, while also detect significant growth changes across the scanning period. Fig 1 shows ultrasonographic images of these pregnancies on each scanning day. The first comprehensive pre-natal study of the ovine embryo was provided by Green and Winters (43), where it was common practice to recover embryos from culled ewes at specific intervals following mating. Studies like the one mentioned were instrumental in creating the foundations for sheep pre-natal development and while this method enabled accurate measurements, sample sizes were often small. Nevertheless, the values recorded in the current study are relatively similar to those obtained by Bryden [60], Schrick [8] and Grazul-Bilska [61], who all reported the crown-rump length of ovine embryos on day 19–20 and 29–30 to be 0.6 and in the range of 1.6–2.0cm, respectively, compared to the 0.4 and 1.4 cm recorded in the current study. With the advent of advanced ultrasonography technologies, size measurements earlier than day 19, like the ones recorded in the current study, can be added to our existing knowledge and will help understand more about the developing ovine embryo.

## Conclusion

The current study has shown that ovine embryos can be detected, with relatively high accuracy, as early as pre-implantation (day 12/14) when using a 10MHz transducer for transrectal ultrasound. Employing this technique, we were able to confirm that the majority of embryonic loss occurs leading up to and during implantation but this was unaltered by the addition of exogenous progesterone on day 3 following AI. However, when exposed to exogenous progesterone as early as day 0, embryo mortality increased. This is thought to be due to the prematurely high levels of progesterone, accelerating the transition and development of the embryo into the uterus. Results from the current study could be used to support the theory that in sheep, the supplementation of exogenous progesterone prior to implantation is ineffective in reducing embryonic loss. Perhaps future attention should be paid to factors occurring prior to fertilisation or even ovulation, to try and identify characteristics of mortality-prone oocytes and embryos that once prevented, could lead to an increase in early embryonic survival and a reduction in reproductive wastage for the Australian sheep industry.

## Supporting information

S1 Table Transrectal ultrasound (Esaote Germany, MyLab™One VET, equipped with electronic linear array 10–5 MHz transducer (SV3513 Vet)) images showing measured structures on Day 10, 12, 14, 17, 19 and 28. (PDF)
