# Supplementary material for: Using transrectal ultrasound to examine the effect of exogenous progesterone on early embryonic loss in sheep
Source: PLoS One. 2017 Aug 25;12(8):e0183659. doi: 10.1371/journal.pone.0183659 (PMC5571956; doi:10.1371/journal.pone.0183659)
Supplement: S1 Table — (PDF) [file pone.0183659.s001.pdf]

Image ref	Image	Measured structure	Size (mm)
A	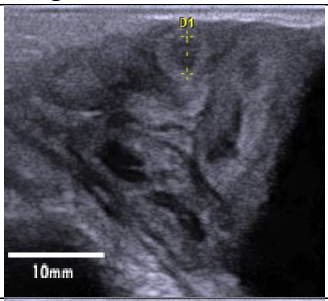	Endometrium height measured on Day 10 (D1)	D1=4.12
B	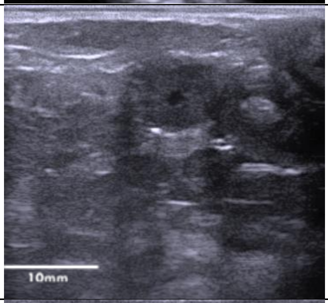	2 Corpora lutea present on the ovary, measured on Day 10 (D1 and D2)	D 1 x 1.18 mm<br>D2 x 1,48 mm
C	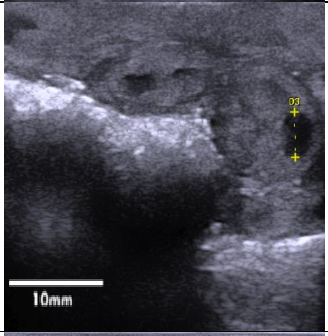	Trophoblastic expansion measured on Day 12 (D3)	D3= 6.68
D	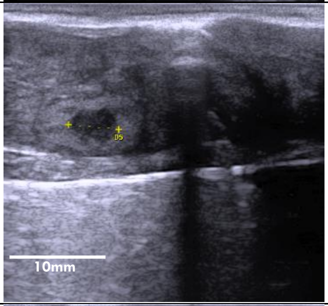	Trophoblastic expansion at Day 14 (D5)<br>Embryonic vesicle	D3=7.92
E	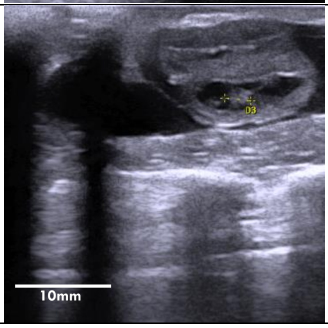	Embryo at Day 17 (D3)	D3= 4.14

F	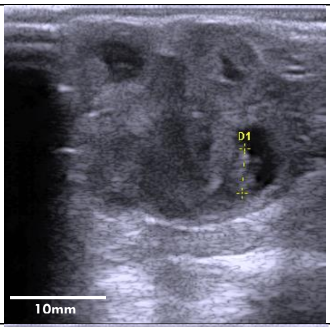	Embryo (D1) clearly visible within embryonic vesicle, measured on Day 19	D1=5.46
G	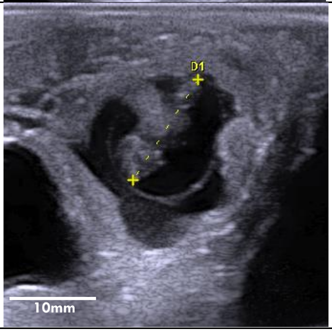	Embryo (D1) surrounded by amniotic membrane, measured on Day 29	D1=13.9
